# Changing Hydrozoan Bauplans by Silencing Hox-Like Genes

**DOI:** 10.1371/journal.pone.0000694

**Published:** 2007-08-01

**Authors:** Wolfgang Jakob, Bernd Schierwater

**Affiliations:** 1 Division of Ecology and Evolution, Stiftung Tieraerztliche Hochschule Hannover, Hannover, Germany; 2 Department of Molecular, Cellular and Developmental Biology, Yale University, New Haven, Connecticut, United States of America; University of Cape Town, South Africa

## Abstract

Regulatory genes of the Antp class have been a major factor for the invention and radiation of animal bauplans. One of the most diverse animal phyla are the Cnidaria, which are close to the root of metazoan life and which often appear in two distinct generations and a remarkable variety of body forms. Hox-like genes have been known to be involved in axial patterning in the Cnidaria and have been suspected to play roles in the genetic control of many of the observed bauplan changes. Unfortunately RNAi mediated gene silencing studies have not been satisfactory for marine invertebrate organisms thus far. No direct evidence supporting Hox-like gene induced bauplan changes in cnidarians have been documented as of yet. Herein, we report a protocol for RNAi transfection of marine invertebrates and demonstrate that knock downs of Hox-like genes in Cnidaria create substantial bauplan alterations, including the formation of multiple oral poles (“heads”) by *Cnox-*2 and *Cnox-*3 inhibition, deformation of the main body axis by *Cnox*-5 inhibition and duplication of tentacles by *Cnox-*1 inhibition. All phenotypes observed in the course of the RNAi studies were identical to those obtained by morpholino antisense oligo experiments and are reminiscent of macroevolutionary bauplan changes. The reported protocol will allow routine RNAi studies in marine invertebrates to be established.

## Introduction

RNAi and morpholino antisense studies on regulatory genes have offered crucial insights into the genetic mechanisms underlying bauplan changes in metazoan animals [e.g. 1]–[8]. It is highly unfortunate that only a very limited number of gene silencing studies in basal metazoan lineages have yet been possible due to methodological limitations resulting from working with multicellular marine animals [5], [7]–[10]. Functional studies on regulatory genes in the Cnidaria are particularly desirable as the diversity of bauplans within the phyla is one of the richest. Cnidaria harbor a minimal set of 5 (e.g. the hydrozoan *Eleutheria*) to 10 (e.g. the anthozoan *Nematostella*) Hox-like genes [11], [12] and expression studies have shown that these genes are regionally expressed along the aboral-oral axis [e.g. 13]–[17]. Some of these expression studies on homologous Hox-like genes revealed different expression patterns in different species and have led to incongruent results [11]. Functional studies may help to resolve these inconsistencies and learn about the function of Hox-like genes in early metazoan evolution. We here report a useful means for gene silencing by RNAi in marine invertebrates, validated with the use of several controls, including morpholino antisense oligos and information from expression data, demonstrate gene specific effects. This initial report will be followed by further in depth studies of the expression and function of all genes in each developmental stage of the complex 3-stage metagenic life-cycle of *Eleutheria dichotoma*.

## Results and Discussion

**Figure 1:**
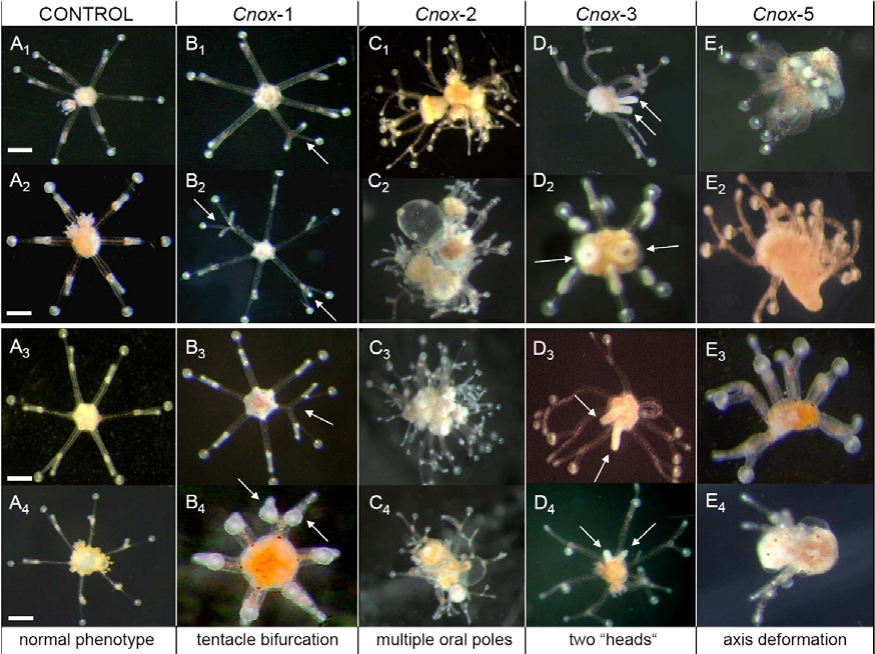
Observed phenotypic changes in knock down experiments of Hox-like genes in the hydrozoan *Eleutheria dichotoma.* Four of the five Hox-like genes produced phenotypically abnormal medusae (PAMs) in gene knock down studies. The main features of bauplan change relate to additional tentacle bifurcation (*Cnox*-1; see arrows), multiple oral poles or heads (*Cnox*-2), head duplication (*Cnox*-3; see arrows), and oral-aboral body axis deformation (*Cnox*-5). The two upper panels show life pictures of medusae transfected with double stranded RNA (B_1,2_, C_1,2_, D_1,2_, E_1,2_), and the lower panel medusae transfected with morpholino oligos (B_3,4_, C_3,4_, D_3,4_, E_3,4_). A_1,2_ and A_3,4_ are controls, scale bar is 100 µm. While we here unambiguously show that Hox-like genes can be silenced we think it would be premature to derive final conclusions on their functions yet.

**Figure 2:**
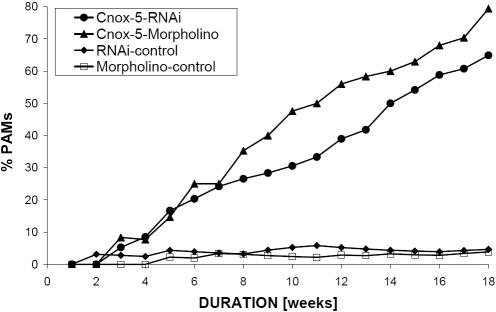
Time course for the development of phenotypic effects resulting from Cnox-5 gene knock down. Both morpholino oligo and RNAi experiments result in an increase of phenotypically abnormal medusae (PAMs) over time. Shown is the time course of increasing numbers of PAMs as a result of inhibition by double stranded RNA and inhibition by morpholino-antisense oligonucleotides. In each case control 1 is untreated animals cultured in normal seawater, and control 2 is a sense morpholino oligonucleotide or dsRNA targeted to the *Trox*-2 gene from the placozoan *Trichoplax adhaerens* respectively. Independent controls were run for each single experiment.

In addition to three previously cloned Hox-like genes, *Cnox*-3 to *Cnox*-5 [18], we also isolated full-length cDNA sequences of *Cnox*-1 and *Cnox*-2 from the hydrozoan *Eleutheria dichotoma*. We have developed a protocol enabling RNAi studies on marine invertebrates and performed different gene inhibition studies using RNAi and morpholino antisense oligos for all five genes. Based on partial and full length homeobox sequences, all five Cnox genes show a clear relationship to Antp class genes [18]. The full length cDNA sequences support the previous suggestion, as well as the recent view that the Cnidaria possess Hox related genes (Hox-like) [12]. All Cnox genes are relatively short, harboring coding sequences of between 684bp (*Cnox*-3-Ed) and 924bp (*Cnox*-1-Ed; see suppl. data, Fig. S1). Outside the homeobox, no similarity to Hox or other Hox related genes is seen, except for the presence of a heptapeptide (RELENRR) in *Cnox*-2, also found in an orthologous gene from *Cassiopeia xamanchana* [19] (for a possible derived amino terminal HEP-motif see [20], [21]). No other conserved motifs were found outside the homeobox, making the design of specific antisense oligos and dsRNA unproblematic (see suppl. data Fig. S1). In several repetitions vegetative medusae of *E. dichotoma* were subjected to gene silencing experiments (see Fig. S2). Transfected medusae of the hydrozoan *Eleutheria dichotoma* showed remarkable alterations in morphology if *Cnox*-1-Ed, *Cnox*-2-Ed, *Cnox*-3-Ed or *Cnox*-5-Ed were inhibited (see Fig. 1 and Tab. 1; [e.g. 5, 13, 22]). The most striking knock down phenotype is medusae developing multiple oral poles or “heads”. This phenotype regularly developed as a result of inhibition of the *Cnox*-2 or *Cnox*-3 gene, or inhibition of both together. If medusae developed two manubriae (“heads”), both were fully functional and capable of independent feeding. More than two manubriae (i.e. multiple heads) were regularly observed when *Cnox*-2 was inhibited. Another striking phenotype from the same inhibition experiment relates to a massive deformation of the primary body axis, the oral-aboral axis. Budding medusae did not develop defined aboral and oral body poles; the tentacles, however, were well developed. Identical phenotypes were regularly observed also when *Cnox*-5 was inhibited (up to 80% PAMs [**p**henotypic **a**bnormal **m**edusae] after 18 weeks). A third type of phenotype resulted from the inhibition of *Cnox*-1, which predominantly created additional tentacle bifurcations. Remarkably all of the above described, RNAi driven phenotypes were also observed when the same genes were inhibited by morpholino antisense oligos (Fig. 1). The latter were more effective inhibiting single or combinations of Cnox genes, while qualitatively no differences were detected between RNAi and morpholino oligo inhibition experiments. In morpholino oligo studies the percentage of PAMs was significantly higher (Fig. 2). This observation is consistent with former observations [23], [24]. The findings that both, morpholino and double stranded RNA inhibition, resulted in the same phenotypes, suggests specific and successful inhibition of the target genes, and indicates that the observed phenotypic changes are not due to morpholaxis or regeneration processes. In more than five decades of experimental research on *Eleutheria* medusae, none of the observed phenotypes–with one exception-has ever been seen in medusae under a variety of favorable and unfavorable physiological conditions [25]–[28]. Only tentacle deformations were occasionally seen (but never exceeding more than 1% in a population; Carl Hauenschild, pers. comm.). The presence of supplementary mouths or bifurcated tentacles can be normal in some other hydrozoans (Ferdinando Boero, pers. comm.). Interestingly Bouillon et al. (1997; [29]) and Boero et al. (2006; [30]), suggested that multiple manubriae in the senescent medusa of *Codonorchis octaedrus* might be due to the activation or repression of genes leading to clonal morphs. Since medusa buds develop from a very small number of epithelia and undifferentiated I-cells, it seems likely that transfection manifests here and subsequently enters the majority of cells in the daughter medusae. Here transfection of neighboring cells would be very efficient [31]. In future studies, it will be interesting to examine the potential for overlapping functions of these Hox-like genes. It would also be desirable to perform silencing studies of *Cnox*-1 to *Cnox*-5 genes on earlier developmental, i.e. larval stages. However, this would clearly exceed the scope of this paper.

**Figure 3:**
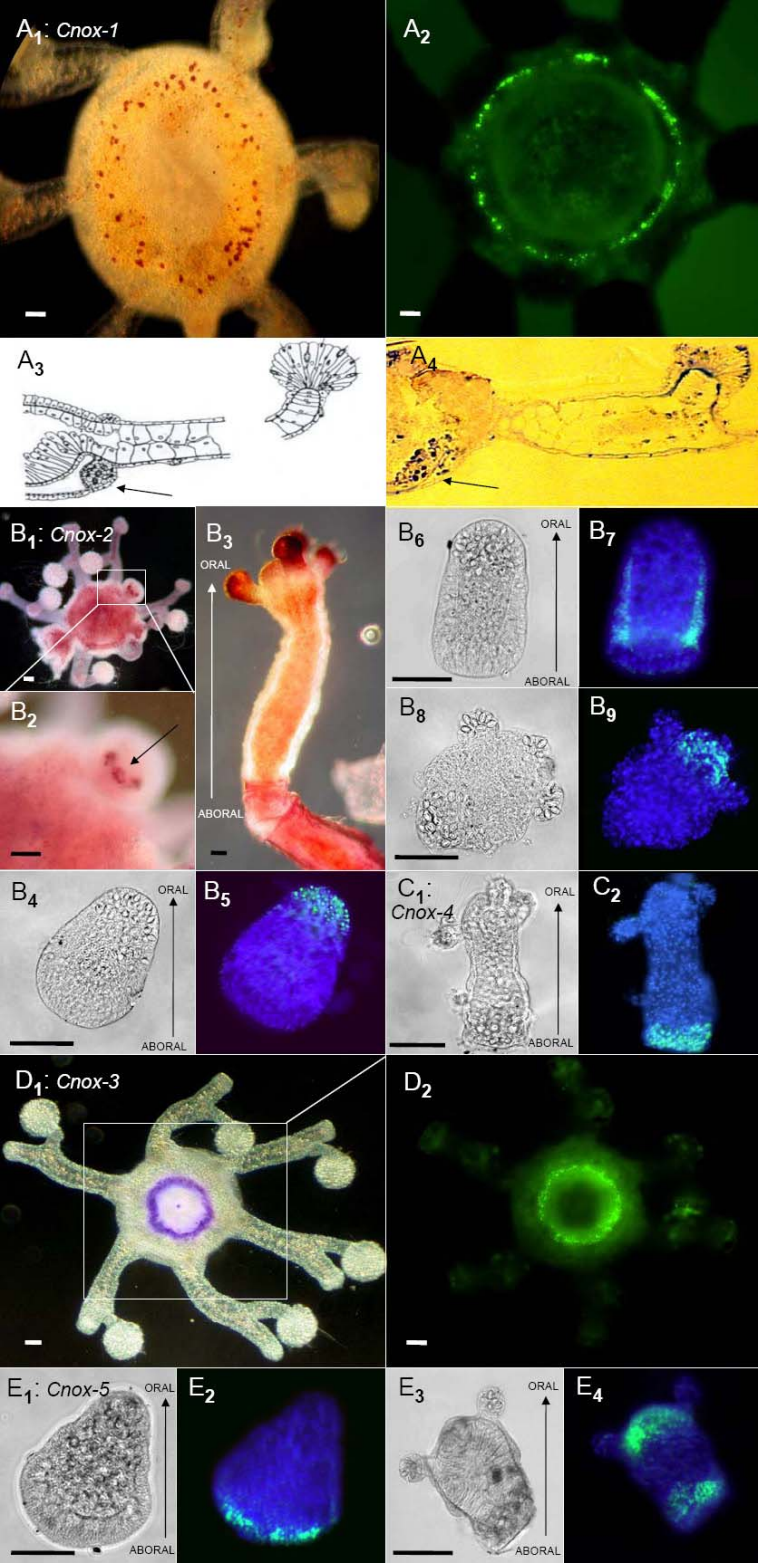
In situ expression of Hox-like genes in the hydrozoan *Eleutheria dichotoma.* The five Hox-like genes, *Cnox-*1 to *Cnox-*5, display differential spatio-temporal expression patterns along the oral-aboral body axis. *Cnox-*1 (A_1_–A_4_) is expressed ectodermally in the so-called “Nesselring”, an area of undifferentiated cells lining the ring canal of medusae (cross section: A_3_, A_4_). *Cnox-*2 is expressed in the entoderm of developing medusa buds (B_1_, B_2_), in 2-day-old planula larvae ectodermally and orally (B_5_), in 5-day-old planula larvae along the body column ectodermally (B_7_) and at the oral pole of primary (B_9_) and adult polyps (B_3_; single polyp with 4 tentacles). *Cnox-*4 is exclusively expressed at the aboral pole of primary polyps (C_2_). *Cnox-*3 expression perfectly marks the most ectodermal oral part of the manubrium (D_1_, D_2_). *Cnox-*5 shows a remarkable pattern of expression moving from aboral only in the planula larva (E_2_) to both oral and aboral simultaneously in the metamorphosing polyp (E_4_). NBT/X-phosphate (A_1_, A_4_, B_1_–B_3_, D_1_) and fluorescein-labeled probes (A_2_, B_5_, B_7_, B_9_, C_2_, D_2_, E_2_, E_4_). Signals in B_5_, B_7_, B_9_, C_2_, E_2_ and E_4_ are overlaid with DAPI staining. Morphologies are shown in light microscopy (B_4_, B_6_, B_8_, C_1_, E_1_, E_3_). Scale bar is 50µm. A_2_, D_1_, E_1_–E_4_ are reprinted with permission from Elsevier Publishers [11].

With respect to gene expression, all five Cnox genes revealed *cum grano salis* unambiguous patterns (Fig. 3). *Cnox*-1 and *Cnox*-3 were expressed in the medusa generation only, while *Cnox*-4 and *Cnox*-5 were detectable in the polyp generation (including planula larva) only. *Cnox*-2 was the only gene which was expressed in all life cycle stages; planula, polyp, and medusa (see table 1). Relating the observed knock down phenotypes to expression profiles leads to some intriguing speculations regarding possible gene function. Oral ectodermal expression in the cnidoblast channel directly suggests a role for *Cnox*-1 in tentacle formation and regeneration. The cnidoblast channel harbors differentiating cnidocytes and undifferentiated totipotent cells, both of which are essential for tentacle development [25]. A possible function of *Cnox*-2 in axis formation has previously been suggested before, and is supported by both the multiple oral head phenotypes (resulting from gene knock down) and the observed entodermal expression pattern in early medusa buds, where oral-aboral axis formation initiates. In the absence of *Cnox*-2 expression, either regulation of the aboral pole or a head inhibitor is missing [14], [15], [32]–[34]. As a consequence several oral poles (heads) develop, and orient themselves to the single aboral pole. A complementary function seems suggestive for the *Cnox*-3 gene, and it may play a crucial role in oral-aboral pole formation later in ontogeny. In adult medusae it is expressed orally in the ectoderm and possibly inhibits the formation of additional oral poles. When inhibited, a second oral pole can develop in adult medusae (although note that more than two oral poles have not been observed in *Cnox*-3 studies). For *Cnox*-5 we suggest a more upstream regulatory function. Although this gene is expressed at a very low level (detectable by semi-quantitative PCR but not by *in situ* hybridization; see suppl. data, Fig. S3), inhibition of this gene results in the highest number of PAMs; more than 80% of medusae show a dramatically deformed oral-aboral axis structures, except for the tentacles which develop normally. The observation that *Cnox*-5 produces a robust phenotype but not a visible *in situ* signal seems puzzling. We may conclude that expression is very low and possibly limited to a small number of undifferentiated I-cells. Alternatively, we can not exclude that *Cnox*-5 may be expressed in only a very short time window in the medusa stage. The expression of the *Cnox*-5-Ed gene of *Eleutheria*, an orthologue of both *Anthox*-6-Nv (*Nematostella*) and *Cnox*-1-Pc (*Podocoryne*; see table 2), may provide a clue. Indeed, the expression of this gene is dynamic during the development of *Eleutheria*, moving from the aboral (like *Podocoryne*) to the oral pole (like *Nematostella*). Hence a heterochronic change in the regulation of this gene might explain the evolution of its expression in the Cnidaria. It is an interesting speculation that *Cnox*-5 might be involved in the regulation of *Cnox*-2 and *Cnox*-3 in the medusa generation. Any further conclusions would be premature and clearly exceed the scope of this paper.

**Table 1:** Cnox gene expression and knock down phenotypes in the hydrozoan *Eleutheria dichotoma*.

Cnox-gene	*In situ* expression	Dominant knock down phenotype
*Cnox*-1	Medusa: + ; ectodermal, oral ring in the “cnidoblast channel”	>71% of medusae with abnormal tentacle structures
	Planula: − ;	
	Polyp: − ;	
*Cnox*-2	Medusa: + ; in early buds, entodermal	>66% of medusae with multiple oral poles
	Planula: + ; ectodermal, oral	
	Polyp: + ; in polyps and primary polyps	
	ectodermal, oral	
*Cnox*-3	Medusa: + ; ectodermal, oral ring around the manubrium	>60% of medusae with double oral pole (two heads)
	Planula: − ;	
	Polyp: − ;	
*Cnox*-4	Medusa: − ;	No visible phenotypic effect
	Planula: − ;	
	Polyp: + ; in primary polyps ectodermal, aboral	
*Cnox*-5	Medusa: − ;	>79% of medusae with deformed aboral-oral body axis
	Planula: + ; ectodermal, aboral.	
	Polyp: + ; ecto- and entodermal, oral and aboral	

Explanation is given in the text.

**Table 2:** Homology nomenclature and expression of Hox/ParaHox-like genes in Cnidaria.

GENE	ORGANISM	EXPRESSION	REFERENCE
***Cnox*** **-1-Ed** Hox-like	*Nematostella vectensis*; *Anthox*-1	**polyp:** ectodermal&aboral	Finnerty, 1998, 2003; [44], [45]
	*Hydra magnipapillata*; *Cnox*-1	???	Naito et al., 1993; Gauchat et al., 2000; [46], [40]
***Cnox*** **-2-Ed** ParaHox-like	*Hydractinia symbiolongicarpus; Cnox*-2	**polyp:** ectodermal, oral&aboral, throuout the length of the body column	Murtha et al., 1991; Cartwright&Buss, 1999; Cartwright et al., 1999, 2006; [47], [48], [32] [33]
	*Hydra vulgaris; Cnox-*2	**polyp:** suppressed oral^1^; body column^1,2^; ectodermal & oral^2,^	Endl et al., 1999^1^; Gauchat et al., 2000^2^; [34], [43]
	*Chlorohydra viridissima; Cnox*-2	**polyp:** ectodermal&oral (high level)	Schummer et al., 1992; [49]
	*Nematostella vectensis*; *Anthox*-2	**polyp:** ectodermal&oral	Finnerty et al., 2003; [45]
	*Acropora millipora; Cnox-*2-Am	**polyp:** ectodermal&oral	Hayward et al., 2001; [50]
		**planula:** ectodermal&vegetal (oral)	
	*Hydra magnipapillata*; *Cnox*-2	**polyp:** ectodermal&oral (low level), aboral (high level); body column	Senk et al., 1993a, b; [13], [14]
	*Podocoryne carnea; gsx*	**polyp:** ectodermal&oral	Yanze et al., 2001; [16]
		**early medusa:** ectodermal&oral; **adult:** entodermal; **planula:** entodermal&animal (aboral)	
***Cnox*** **-3-Ed** Hox-like	*Hydra vulgaris; Cnox-*3	**polyp:** ectodermal&oral	Gauchat et al., 2000; [43]
***Cnox*** **-4-Ed** ParaHox-like	*Metridium senile; Anthox*-4	???	Finnerty&Martindale, 1997; [51]
***Cnox*** **-5-Ed** Hox-like	*Podocoryne carnea; Cnox-*1-Pc	**(primary-) polyp:** moves from aboral to oral (same in *Eleutheria*); **medusa:** striated muscle tissue cells; **planula:** ecto-&entodermal animal (aboral)	Aerne et al., 1995; Yanze et al., 2001; Galliot&Schmid, 2002; [52], [16], [53]
	*Hydra vulgaris; Cnox*-1^1,2^; *Cnox*-3^3,4,5^	**polyp:** ectodermal&oral^1,3^; around the hypostom; tentacle zone^2,3^	Schummer et al., 1992^1^; Gauchat et al., 2000^2^; Smith et al., 2000^3^; Bode, 2001^4^; Senk et al., 1993a^5^; [49], [43], [54], [55]
	*Chlorohydra viridissima; Cnox*-1	**polyp:** oral	Schummer et al., 1992; [49]
	*Hydra magnipapillata*; *Cnox*-1	???	Naito et al., 1993; [46]
	*Nematostella vectensis*; *Anthox*-6	**Polyp&planula:** entodermal, oral.	Finnerty&Martindale, 1999; Finnerty, 2003; Finnerty et al., 2004 [56], [45], [17]

Putative homology assignment of Cnox genes according to Gauchat et al. (2000) [43], Finnerty et al. (2003) [21] and Kuhn et al. (1996) [18].

Notably, some of the observed gene inhibition phenotypes look like direct links to the bauplan patterns found in other hydrozoan taxa. The multiple oral poles (or multiple heads) phenotype is strikingly suggestive of colonial hydrozoans, or colonial cnidarians in general. Many colonial cnidarians form colonies by adding oral poles to a common stalk (stolon or hydrocaulus). Each head is functional in feeding and connects to a shared gastrovascular cavity, just as in some *Cnox*-2, *Cnox*-3 and *Cnox*-5 inhibited *Eleutheria* medusae. Other cnidarian bauplans derive from multiplication of tentacle structures or whole tentacles. The already bifurcated tentacles in *Eleutheria* duplicate further if *Cnox*-1 or *Cnox*-2 is inhibited, and the resulting phenotypes are similar to those found in several hydrozoan groups such as *Cladonema radiatum* [35]. As a working hypothesis, it seems to us an intriguing idea that Cnox genes in Cnidaria may provide a very efficient means for macroevolutionary bauplan alteration, for example by multiplication of body parts. The RNAi transfection protocol developed here opens useful and efficient avenues to functional evolutionary genomics not only in Cnidaria, but to basal marine invertebrates in general and probably also to vertebrate larvae.

## Methods

### Animal material

*Eleutheria dichotoma* medusae were collected in Southern France (Banyuls-sur-mer) and have been maintained in cultures for several years under constant laboratory conditions as described earlier [28]. Medusae were fed twice a week with 3–4 days old brine shrimp larvae, *Artemia salina*. 6 hours after feeding and again on the following day, the water was changed. Under laboratory conditions, *E. dichotoma* reproduces alternatively by vegetative budding or bisexual reproduction. For *in situ* hybridization, growing, budding, and sexual medusae, planula larvae, primary polyps, as well as growing and budding polyps were analyzed.

### Cloning and sequencing of *Eleutheria dichotoma* Antp genes

Total RNA was isolated from whole tissue of medusae and polyps using a total RNA-Isolation kit (Promega). First-strand cDNA was synthesized with an oligo (dT)-adapter-primer, reverse transcriptase (Superscript, Invitrogen), and total RNA as template, following the manufacturer's protocol (for detail see [19]). Using sequence information from the homeobox fragments, 3′RACE and 5′RACE [36] were performed to amplify the cDNA ends of the homeobox genes in order to obtain full-length sequences [37].

### *In situ* hybridization

Whole mount *in situ* hybridization followed a slightly modified protocol developed for Placozoa [38]. Deviations from the original protocol include: (i) proteinase K treatment was done for 10 min at 37°C; (ii) no post-fixation in 4% paraformaldehyde/0.2% glutaraldehyde was allowed; (iii) Digoxigenin and fluorescein labeled RNA sense and antisense probes of variable length were synthesized from the PCR amplified 5′ regions of the different genes (*Cnox*-1: 401 bp; *Cnox*-2: 426 bp; *Cnox*-3: 241 bp; *Cnox*-4: 199 bp; *Cnox*-5: 219 bp; see suppl. data).

### Gene inhibition studies

For the gene inhibition experiments replicates each of ten vegetative medusae per experiment were maintained in single culture dishes in a volume of 5 ml artificial seawater.

### a) Cnox gene inhibition by RNAi

Double stranded RNA was synthesized from cDNA templates (cloned into pGEM-T vectors) by using Sp6/T7-RNA polymerase. Single RNA strands of the 5′ ends of the genes (*Cnox*-1: 401 bp; *Cnox*-2: 426 bp; *Cnox*-3: 241 bp; *Cnox*-4: 199 bp; *Cnox*-5: 219 bp) were synthesized at 37°C for 2 h in 20 µl transcription buffer (Roche) in the presence of 250 ng template, 40 U RNase-inhibitor, 20 U T7 or SP6 RNA polymerase, and 10 mM each NTP, including fluorescein UTP. After digestion of the DNA template, ssRNA was precipitated and resuspended in ddH2O, containing 1 μl RNase inhibitor, before complementary strands were annealed. Before transfection with dsRNA, vegetative medusae were gently acclimated from artificial seawater conditions in a stepwise manner, from 35 ppt to 10 ppt salinity. First we added 10 μl FuGENE™-6 (HD)-Transfection Reagent to a total volume of 150 µl of sterile ddH_2_O containing 5 ppt artificial seawater salt (the undiluted FuGENE ™-6 (HD)-reagent may not come into contact with any surface other than the pipette tip). After adding 5 µl of dsRNA (∼5μg; FuGENE-Reagent: dsRNA 2:1, µl and µg respectively), contents were very gently mixed and incubated at 20°C for 45 min. Transfection of animals with dsRNA was done in a total volume of 500 µl for 3 hours. Animals were reacclimatized stepwise back to normal seawater conditions (35 ppt salinity). The first phenotypes were observed 12 days after transfection. By the end of the experiments, i.e. after 18 weeks, 60-80% of all animals showed abnormal phenotypes (*Cnox*-1: 71,3%; *Cnox*-2: 66,4%; *Cnox*-3: 60,3%; *Cnox*-5: 79,7%; N = 90–140 medusae per experiment). In all control populations, the percentage of PAMs was fewer than 3%.

### b) Cnox gene inhibition by morpholino antisense oligonucleotides

For each Cnox gene, a 25 nucleotide long chemically modified morpholino antisense oligonucleotide [Gene Tools, LLC; e.g. 23, 24, 39, 40] was designed, complementary to the 5′ region close to the start methionine codon (suppl. data, Fig. S1). Transfection with morpholino antisense oligonucleotides was done with the aid of EPEI solution (ethoxilated polyethylenimine; Gene Tools, LLC) in a total volume of 10 ml [cf. 24]. Before transfection, animals were acclimated as described above. Modified oligonucleotides were added to the seawater to a final concentration of 1 µM. The first phenotypes were observed two weeks after transfection. By the end of the experiments, i.e. after 18 weeks, 65–88% of all animals showed abnormal phenotypes (*Cnox*-1: 76,8%; *Cnox*-2: 69,4%; *Cnox*-3: 65,1%; *Cnox*-5: 87,7%; N = 95–160 medusae per experiment). In all control populations, the percentage of PAMs was fewer than 2%.

### c) Controls

We used several means to obtain controls for the RNAi and morpholino antisense oligo experiments, including: (i) Transfection success with the described protocol was verified by means of RT-PCR of silenced target-genes. For this 25 *Cnox*-1 to *Cnox*-5 transfected medusae and 25 *Trox*-2 (Hox-like gene from *T. adhaerens,* Placozoa) transfected control medusae were collected two days after transfection. Total RNA was extracted and RT-PCR was carried out according to Li et al. (2000) [41] (see Fig. S3 and Fig. S4; e.g. [40], [42]). (ii) *In vivo* detection of fluorescein-labeled dsRNA and morpholino oligos after transfection using fluorescence microscopy (see Fig. S5; e.g. [38]). (iii) Target gene *in situ* hybridization of knock down animals (see Fig. S6; e.g. [8]). (iv) Usage of two independent inhibition methods (RNAi and morpholino antisense oligomers) giving qualitatively the same results (see Fig. 2). Controls for RNAi comprised (a) untreated animals in normal seawater, and (b) animals transfected with dsRNA of the *Trox*-2 gene of the placozoan *Trichoplax adhaerens*. Control morpholino oligos comprised (a) a random sequence of the same length and (b) the sense sequence of the experimental Cnox gene morpholino oligos (e.g. [40]).

RNAi studies were repeated ten times and morpholino oligo studies six times, according to the design in Fig. S2. All reported results were highly reproducible. The observed phenotypes were qualitatively identical in all experiments and variation in percentage of PAMs was statistically not significant (p≤0.01, t-test, two-sided; in all cases).

## Supporting Information

Figure S1 cDNA sequences for *in situ* probes, and RNAi and morpholino antisense oligo binding sites of all five Hox-like genes from Eleutheria dichotoma. *Cnox*-1 to *Cnox*-5 cDNA sequences from *Eleutheria dichotoma*. Homeodomain sequences are underlined, double-stranded RNA used in RNAi experiments as well as *in situ* probe sequences are highlighted in yellow. The complement of the morpholino antisense oligonucleotides is shown in red.
(0.04 MB PDF)

Figure S2 Experimental design for gene silencing studies on vegetative medusae of the hydrozoan *Eleutheria dichotoma*. At day 0, each of the 10 vegetative i.e. budding medusae were placed in a Boveri dish and treated either with dsRNA (RNAi) or antisense morpholinos, or treated in different ways as controls (Control). After approximately one week, the first medusa buds are released from the parent medusa (circled and highlighted), which then start reproducing vegetatively. In this way, by the end of the experiment the total number of medusae increases to values between 90 and 160 medusae. The original 10 parent medusae at the beginning of the experiment thus represent some 6 to 11% of the final population. If one assumes that only medusa buds are transfected, the percentage of PAMs could be as high as 90% by the end of the experiment.
(0.15 MB PDF)

Figure S3 RT-PCR of all five Hox-like genes in Eleutheria dichotoma. Differential expression of *Cnox*-1 to *Cnox*-5 gene in *Eleutheria dichotoma* medusae. Shown are products of RT-PCR after 27 cycles of *Cnox*-1 (lane 1; 401bp), *Cnox*-2 (lane 2; 426bp), *Cnox*-3 (lane 3; 241bp), *Cnox*-4 (lane 4; 199bp), *Cnox*-5 (lane 5; 219bp) and *Eleutheria*-actin (lane 6; 152bp). *Cnox*-4 and *Cnox*-5 are only weakly expressed.
(0.10 MB PDF)

Figure S4 RT-PCR of *Cnox*-1-Ed after gene knock down. Transfection with dsRNA significantly reduces Cnox gene expression. RT-PCR products from untreated control animals are shown in lanes 1 and 2, from control dsRNA animals in lanes 3 and 4, and from *Cnox*-1 dsRNA-infected animals in lanes 5 and 6. Products in lanes 1, 3, and 5 are actin controls. Note the strong decline of *Cnox* product in lane 6 relative to lanes 2 and 4. Shown here is the example for *Cnox*-1 in *Eleutheria dichotoma*; RT-PCR controls look similar for all five Hox-like genes (data not shown). RT-PCR controls were taken as subsets from the transfected animals used in the gene silencing studies.
(0.10 MB PDF)

Figure S5 *In vivo* detection of fluorescein-labeled dsRNA (A) and morpholino oligomers (B) after transfection.
(0.10 MB PDF)

Figure S6 Target gene *in situ* hybridization of knock down animals. *In situ* hybridization of *Cnox*-1 gene in a *Cnox*-1 knock down animal (A'), in a RNAi-control animal (B), in a Morpholino-control animal (B') and *Cnox*-3 *in situ* hybridization in a *Cnox*-1 knock down animal. Medusa morphology is shown in light microscopy (A) and DAPI staining of the same medusa (A').
(0.29 MB PDF)
