# Supplementary material for: Changing Hydrozoan Bauplans by Silencing Hox-Like Genes
Source: PLoS One. 2007 Aug 1;2(8):e694. doi: 10.1371/journal.pone.0000694 (PMC1931613; doi:10.1371/journal.pone.0000694)
Supplement: Figure S1 — cDNA sequences for in situ probes, and RNAi and morpholino antisense oligo binding sites of all five Hox-like genes from Eleutheria dichotoma. Cnox-1 to Cnox-5 cDNA sequences from Eleutheria dichotoma. Homeodomain sequences are underlined, double-stranded RNA used in RNAi experiments as well as in situ probe sequences are highlighted in yellow. The complement of the morpholino antisense oligonucleotides is shown in red. (0.04 MB PDF) [file pone.0000694.s001.pdf]

cDNA *Cnox*-1

```
AACTCGTCAAATCAATTCATTGTTATAATTATATGCTACAAGATTGTTAGTAAAGATAAT   60

ATAAATATCAAAACTATTCTTAAAAAAGGTAGTTTACTTTCAGCAATTATTTTTTAAATC  120

TATTAATGAATTAGTAAATTAAATACTAATGGAAATTTCTCGTTTGCAACAAATTGATTC  180
                             M  E  I  S  R  L  Q  Q  I  D  S
AATTCAAAATAATGACAATGATGATAGATACCACTATGCACACAATTCAAGAACACCTGC  240
 I  Q  N  N  D  N  D  D  R  Y  H  Y  A  H  N  S  R  T  P  A
TTTGATATCAAGCCACGCTACAAATTTTCGATCTGCTTTTCTCCCAACAACTCTGTCAAG  300
 L  I  S  S  H  A  T  N  F  R  S  A  F  L  P  T  T  L  S  S
TCACCAATATCAAAGTAGCGTTATAAGAAATATCGATAATAGTACGGATACGACAAGTTT  360
 H  Q  Y  Q  S  S  V  I  R  N  I  D  N  S  T  D  T  T  S  F
TGGTTCCTTTAGTCACGGAAATGAAAGTTCAACACACAATAGCTTACCGTATCCTCCGAA  420
 G  S  F  S  H  G  N  E  S  S  T  H  N  S  L  P  Y  P  P  N
CTCGACTCATATCGAACTACCGTCTTATTTATCAAATACAGGTTTACCGCCAGCAACATC  480
 S  T  H  I  E  L  P  S  Y  L  S  N  T  G  L  P  P  A  T  S
TAGTTTATCAGCATTCTATAATCCAATTCATTCTTCGTCGCAAGCACACCTCGCAGAATA  540
 S  L  S  A  F  Y  N  P  I  H  S  S  S  Q  A  H  L  A  E  Y
TCAACAATGGCCGTACGGCAACGTTTCACCTTCTGGGAATTATATATACGGATATGGTAG  600
 Q  Q  W  P  Y  G  N  V  S  P  S  G  N  Y  I  Y  G  Y  G  S
TCCGATGAATAACGGATTCAATCCGTATTCTTCTGTTGATAGTAATGGATTTGCAGGATC  660
 P  M  N  N  G  F  N  P  Y  S  S  V  D  S  N  G  F  A  G  S
GAGTTCCTGGTTATATAGAGATATTGATAGTAAACGCAAACGAATGACTTATTCGAGAAA  720
 S  S  W  L  Y  R  D  I  D  S  K  R  K  R  M  T  Y  S  R  K
ACAGTTATTAGAACTTGAAAAAGAATTCCACTTAAGTCATTTTCTGAAAAAAGAAAGAAG  780
 Q  L  L  E  L  E  K  E  F  H  L  S  H  F  L  K  K  E  R  R
AGTTGACTTGGCAAAACAGCTTAATCTAAGTGAACGTCAAATAAAGATTTGGTTTCAAAA  840
 V  D  L  A  K  Q  L  N  L  S  E  R  Q  I  K  I  W  F  Q  N
TCGTAGAATGAAGTTTAAAAAGGAAAATAAAAAAACTTCCAGTTCTGAATCAATTATGAA  900
 R  R  M  K  F  K  K  E  N  K  K  T  S  S  S  E  S  I  M  N
TCAAGAAGCACCAAAAATTGAAGAACATCCTGATATCATTTCTGAAAATGTAACTGAACA  960
 Q  E  A  P  K  I  E  E  H  P  D  I  I  S  E  N  V  T  E  Q
ACAAGAAACAAATGAAAATATAGAAAATGAAGAAAAAGATAAAAAAATGAATATTCAGCA 1020
 Q  E  T  N  E  N  I  E  N  E  E  K  D  K  K  M  N  I  Q  Q
GATAAATTTGAATGATATGAAAAATTTCATGATGTCACACGTTGCTAGGATTTAAATGAA 1080
 I  N  L  N  D  M  K  N  F  M  M  S  H  V  A  R  I
CTATTTACGTTAATTTGCTTTTCATTTCGATACGATCTTTACCAGAGATAAAAGGTTTAT 1140

CGTACAAGGTACACTTGTATGACAAACAAGTTTTTATTTGCTTGCTTTTTTAAGTAAAAG 1200

TTCATACAGTTCTTCATTTGCGAAAAAAAACAAAAATAAAATGAATTTGCGATTTTACAA 1260

ATTTAAAAATATGAAAGGTTTCAACTTCGTTGCTGTTATGAAATAATCTATATGTATATA 1320

CAAAAATTATTTACAACGCTTTAGAAAAAAATAAACTTGCAAATAATAATTACACAAAAT 1380

TTAAAAAAAAAAAAAA                                             1396
```

cDNA *Cnox*-2

```
CATTTGATTACAACAGTTGTCAAAGAAGGAAGAAATAGTTCGATTGAACAAATATTAGTG   60
H   L   I   T   T   V   V   K   E   G   R   N   S   S   I   E   Q   I   L   V
AAAAAAAAAGATATGTCGACTTCAAAGTCGTTTCTTATCGATACAATTATGCATGAAAAA  120
K   K   K   D   M   S   T   S   K   S   F   L   I   D   T   I   M   H   E   K
CAGAAACTATTATTACGTCAGCAACAACCGCTGCACCTTACGTCAAGGGAATCTTCTCCA  180
Q   K   L   L   L   R   Q   Q   Q   P   L   H   L   T   S   R   E   S   S   P
GTTTCAGACCATTCTACTTCAAGATTGATGATGTCATCAGAAGATATTAGGTATAACAGA  240
V   S   D   H   S   T   S   R   L   M   M   S   S   E   D   I   R   Y   N   R
TCCCCTGGTTCTTCGCCTCGTACTCCACCAACACCTCAAGTTATGGACTTTCACATGATG  300
S   P   G   S   S   P   R   T   P   P   T   P   Q   V   M   D   F   H   M   M
GAAAGATCAAGAGCTATTGCATATTGTAATATGAGATGTTCAAGCATTTGTGCAAGCTGT  360
E   R   S   R   A   I   A   Y   C   N   M   R   C   S   S   I   C   A   S   C
CCCCCACACCCTGGTCAACCGATTTGTACTTTGTGTGAACCGAGAGAGGGAGAAGCTGCG  420
P   P   H   P   G   Q   P   I   C   T   L   C   E   P   R   E   G   E   A   A
CCATCTCAACACATGTATACATATTCAAGAGAACCATGTACTAGACACCCGTTTTCAATG  480
P   S   Q   H   M   Y   T   Y   S   R   E   P   C   T   R   H   P   F   S   M
AATCGCGGATCTGCTCGACCTTTAAACCCCCTCACCTCATCTTCAAGACTTCAACAGATA  540
N   R   G   S   A   R   P   L   N   P   L   T   S   S   S   R   L   Q   Q   I
TCCCCAACTTTTGTGCGTGAATTGGAAAATCGGAGAATGCGTTCACAACATGAACACGAA  600
S   P   T   F   V   R   E   L   E   N   R   R   M   R   S   Q   H   E   H   E
CAACAGATGTGCTCTAAATCTAAACGCATACGAACCGCATACACGAGTATTCAGTTGTTA  660
Q   Q   M   C   S   K   S   K   R   I   R   T   A   Y   T   S   I   Q   L   L
GAACTAGAGAAAGAATTTCAAAATAATCGATATCTGTCTAGATTAAGACGTATACAAATC  720
E   L   E   K   E   F   Q   N   N   R   Y   L   S   R   L   R   R   I   Q   I
GCTGCCATGCTAGACTTAACAGAAAAGCAGGTTAAAATTTGGTTCCAGAACAGGAGAGTA  780
A   A   M   L   D   L   T   E   K   Q   V   K   I   W   F   Q   N   R   R   V
AAATGGAAGAAAGATAAAAAAGGCTCGCATGGTTATTCCCCGAGCTCACCAATCAATAGC  840
K   W   K   K   D   K   K   G   S   H   G   Y   S   P   S   S   P   I   N   S
CCGGAATCTCCCATGTAAAAATAAACAGGAGAAATTGAAAAATACTCATATATTCAAACA  900
P   E   S   P   M
TTATTTCGTTTAAATCAAAATAGAAAATTCTTTCTTGAAATACTATATTCTTTATTAACA  960

ATATGTTAAAATTTTTCTAATCGCTAACAAATGAGATTTGGAATTTTATTTATGCATTTC 1020

TGACAAGCTCCAGTTGACATAAAGATAATCTATATTAATCAGAAACCTATTCATAAAACA 1080

CTTTGCATGTCAAAAAAAGTGTAACGCGTTTTTAATTTCAATCGTTAATTTAGTTTAAGT 1140

AAAAGTTTAGCACTCATCACTAGTGCCAAACCTTTTTTTAAGAGTTATAAAGTTACTCAG 1200

TTATAATTTATTAAATGATCTCCTTTGCAGAGACCTATTCTATTATGTTCGGTTTGAATT 1260

TGTAAATATAGCACTTGATATAATTTATTTATTTATTTGCCAATTTATATACATTTCTAT 1320

ACAACAATTATAATATTCAAATAAAATTTAAAATATATATTAATCTAAAAA           1371
```

<u>cDNA *Cnox*-3</u>

```
ACAGATAATACACAAACTAATCTTGAGTCACTGTGTATAAGGTTTCATGATATTTAGATA  60

GAAAAATCTTGAATAATTATCAAAACATAATAAACATCAATTCAAAAAAATTATTTTTCA  120

CTGATGAATTCATTTAATTTTGAAGATAATCTTAATAAAGATGCATTGTATAATCGCCAT  180
   M  N  S  F  N  F  E  D  N  L  N  K  D  A  L  Y  N  R  H
CCTCATGTTCAGAATTATTCTTATCTATCAATAAATTGTAAACAACCAAATGATGATGCG  240
P  H  V  Q  N  Y  S  Y  L  S  I  N  C  K  Q  P  N  D  D  A
TATGTGTATAATAGACAACGTGTTCCAAGCTCTCCAGAGATTCACGGTGATACTAGAATG  300
Y  V  Y  N  R  Q  R  V  P  S  S  P  E  I  H  G  D  T  R  M
GAGCAATTGAGTCAACGTTCATACCAGACATTACAAAGTCGAAGCCATGAAACAGAAGCT  360
E  Q  L  S  Q  R  S  Y  Q  T  L  Q  S  R  S  H  E  T  E  A
CCCGATTATTATTCATATGAGCATATTGATGATGCAAACAAAACGCTAAACTATCATTAT  420
P  D  Y  Y  S  Y  E  H  I  D  D  A  N  K  T  L  N  Y  H  Y
ATGAATAAAAATACTTACGATAATAATACATGGCAAAACAATACACCTCTATCTTGGCAT  480
M  N  K  N  T  Y  D  N  N  T  W  Q  N  N  T  P  L  S  W  H
CATAATATAACATCTCAGCCAGCAGCTTGGCATTCAACGCAAACTAATACCACACCCCAA  540
H  N  I  T  S  Q  P  A  A  W  H  S  T  Q  T  N  T  T  P  Q
TTGGATCAAGGTTATATGTCTTGGAATTGTTGCTATGTTGACAGTAAACGGAAAAGAACT  600
L  D  Q  G  Y  M  S  W  N  C  C  Y  V  D  S  K  R  K  R  T
TCATATTCGCGTAGACAAATATTTGAACTTGAAAACGAGTTTAATAGAAGTCGTTACATC  660
S  Y  S  R  R  Q  I  F  E  L  E  N  E  F  N  R  S  R  Y  I
ACACGTGAAAAAAGAATAGAATTATCCATGATATTAAACTTAACGGAGAGACAAGTTAAA  720
T  R  E  K  R  I  E  L  S  M  I  L  N  L  T  E  R  Q  V  K
ACATGGTTTCAAAATCGTAGAATGAAAACAAAGAAAGAAAAGACAATGGATGATACTTCT  780
T  W  F  Q  N  R  R  M  K  T  K  K  E  K  T  M  D  D  T  S
AATGACTGTTCGATTAAAAGTAAAACTTAAATATCAATAACACTTCAATGAAAAATTATA  840
N  D  C  S  I  K  S  K  T
AAGAATGAATGGAAAATACGAAAGCTTAACTACTGCATACAAATGTAACGCAATTGTTGT  900

TATAGATCATTTTACGTGAAAGTTTAAGGTAACTAAAAAAAAAA                  943
```

cDNA *Cnox*-4

```
TACGGAATAGTTGATAAATTTTTTTCTCAAAATGATTGATACAACACTCAACACGATGCT   60
                                M  I  D  T  T  L  N  T  M  L
GTATTGGGACAACAAATCTTTAGATATTTTTGAAGACAAAAGTGGATTGGGGTTTGAAGT  120
 Y  W  D  N  K  S  L  D  I  F  E  D  K  S  G  L  G  F  E  V
GAACAACTATTTCACAACTAAACATCAATACAACAACCTATCTTATCCAAACTTCATCAA  180
 N  N  Y  F  T  T  K  H  Q  Y  N  N  L  S  Y  P  N  F  I  N
TGATGACAATATAAAGTGTAGCTTAACTCAAACACAAAGTATCTCGAAATCTCCTAAAAG  240
 D  D  N  I  K  C  S  L  T  Q  T  Q  S  I  S  K  S  P  K  S
TGCAGAGATTACTGTCAATAAATTGCAACACGAACAAGACAACTTAATCCAACAACAAAT  300
 A  E  I  T  V  N  K  L  Q  H  E  Q  D  N  L  I  Q  Q  Q  I
TCAAGACCGTACAATTTCATTAACAAATATGAACAACCCCCTCACGAGCAGTTGGCCAAC  360
 Q  D  R  T  I  S  L  T  N  M  N  N  P  L  T  S  S  W  P  T
TTATAATCCCTTACCAAGAACAAACATATTGATGAATGATCCATCAACATCAACATCTTT  420
 Y  N  P  L  P  R  T  N  I  L  M  N  D  P  S  T  S  T  S  F
TACAACTACTTCCCTTTCCCCATGTATGCTTACTCCGCAAGCGAGCGTGCAATGGTTTAA  480
 T  T  T  S  L  S  P  C  M  L  T  P  Q  A  S  V  Q  W  F  N
CCGATCCAACATGGTAAATGATCCTTGGGCTATGGAATATGGGCATGATCCTGCCATGCG  540
 R  S  N  M  V  N  D  P  W  A  M  E  Y  G  H  D  P  A  M  R
AAGCAGACCATGTTTTTCATCTCATCAGACAAGAGAGCTTGAGAAGGAGTTTTTGGTTTG  600
 S  R  P  C  F  S  S  H  Q  T  R  E  L  E  K  E  F  L  V  C
TCAATATGTCACTCGCAGGCGACGGATCGAACTTGCATTTTCACTTAATCTATCTGAGAA  660
 Q  Y  V  T  R  R  R  R  I  E  L  A  F  S  L  N  L  S  E  K
ACAAATAAAAACATGGTTTCAAAATCGCAGAGTGAAAGAAAGAAAACAAAAGAAATGTTC  720
 Q  I  K  T  W  F  Q  N  R  R  V  K  E  R  K  Q  K  K  C  S
AAGTGAAAGTGAACGGTCGGCTTCAAAAATATGAAAAAGAAAACGTTTCTTGAAGAACAA  780
 S  E  S  E  R  S  A  S  K  I
TAGCCTCTTCTGTTTTCGATAAAAAATGGAGCTAGTTGAGGAGACGTAGATGGAAGGTTG  840

ATATCCGTTGATAGAGACATATGGTATCTGAAAATAAAATTTATCTGTCATCACAAGGAC  900

GTAATTACGAACAAAAAGATTATCCTCGATGAACGCAACCATATGTTGATGAAACCGCAA  960

TGCATATGATATCTTTCACTTCAAAGTGTTTTTCCTTCAAAATCAATAAACTTGTTTGAT 1020

CTTTGTTGACAAAACAGTAATCCAATATGTGCTTTAAATTGTTTTTCTTATTTCAATACT 1080

TCCGTTTGTAATTACAAAGAGAGTTTGTAAAACTGTTAAAGTGCTTGCTTATAACACGCC 1140

ATATCAAACGCAGAGTAGAAAAAAAATTCAATATCTTTATCAAAAAAAAAAAA        1193
```

cDNA *Cnox*-5

```
TGACAACATAAGAGACTATATATAAGTAACATGGCAGAGATTGAAAAAGATTTTTTCGAC   60
                              M  A  E  I  E  K  D  F  F  D
TTAATTAATTCAAATAATGAATCCATCGATGTTGAAAGGAAAAATTTAAATGACAAAGAA  120
L  I  N  S  N  N  E  S  I  D  V  E  R  K  N  L  N  D  K  E
ATTAACCCAATGCATACAGCATGTCGGTACTCTAGTCAACATACATATTCTCATACTTTT  180
I  N  P  M  H  T  A  C  R  Y  S  S  Q  H  T  Y  S  H  T  F
GAATATTTCCCAGGAGATTCTCATGTACAGAAAATAAAAAAAGATGATCAAGCTGCACCT  240
E  Y  F  P  G  D  S  H  V  Q  K  I  K  K  D  D  Q  A  A  P
ATACAACTTCCTTGCATGAGCAGTTATTTTAGTAAACCTGGAGAAGTAACTGATGGTTTT  300
I  Q  L  P  C  M  S  S  Y  F  S  K  P  G  E  V  T  D  G  F
GAAGATGCGTTTAAAAAGAGAGATTCGCAAAAGAGATGTTGGAACACCGATAACTTCAAA  360
E  D  A  F  K  K  R  D  S  Q  K  R  C  W  N  T  D  N  F  K
TGGATAAATATTAAACGTGAAAAACGGGCAGCGGATGTTGAATCTACTCAAGATAATCAA  420
W  I  N  I  K  R  E  K  R  A  A  D  V  E  S  T  Q  D  N  Q
AAACAGAGAGAGTTGCACTCGAAGGATATTTGTAAAAAACGTGTATGTTTCACACAAAAA  480
K  Q  R  E  L  H  S  K  D  I  C  K  K  R  V  C  F  T  Q  K
CAGATCGTCGAACTTGAAAAAGAATTTCATTACAATCGATATCTTACACGTGCTCGGAGA  540
Q  I  V  E  L  E  K  E  F  H  Y  N  R  Y  L  T  R  A  R  R
GTAGAGATCGCACAATTGTTAAAACTTACTGAAGCTCAAATAAAAATATGGTTTCAAAAT  600
V  E  I  A  Q  L  L  K  L  T  E  A  Q  I  K  I  W  F  Q  N
CGTCGCATGAAGCAAAAACGAGAACAAAAAGATATGGCAACACCGCATTTTCTTGATAAT  660
R  R  M  K  Q  K  R  E  Q  K  D  M  A  T  P  H  F  L  D  N
CAACTCAATCAGTGCGCAGTTGACTACACGACTTCCTGCCCAGTTAACTTCCCAACTTCA  720
Q  L  N  Q  C  A  V  D  Y  T  T  S  C  P  V  N  F  P  T  S
TGTCCAGTCGAATTCACAAACAATAGAGCATGGTACAATGCTTGTAATCAAGGAGATTCA  780
C  P  V  E  F  T  N  N  R  A  W  Y  N  A  C  N  Q  G  D  S
TCTCATTTTCAATCAATCACTTTTCATTGATCTAATTGAATCGGTATTTACAGGTCAATG  840
S  H  F  Q  S  I  T  F  H
ATTTGTTTGTAATGTGATAGAATATTTCTCCAAAACTTCGAATTATCTGATATATTATTT  900

AAACCATAGTCTATGAGAAATAAGAAAATGAATAAACAAACTGCAGCTCCTTTGAATATC  960

TATTTATTTAAAAATAGAAGAAAACTGAAAATAATAGACTTTTAATTGTAAATATATTAG 1020

TTTATGAATCTTCGCTTTATTTGAAATATATTATTATCTTTAAAAAAAAA           1070
```
