# Supplementary material for: Changing Hydrozoan Bauplans by Silencing Hox-Like Genes
Source: PLoS One. 2007 Aug 1;2(8):e694. doi: 10.1371/journal.pone.0000694 (PMC1931613; doi:10.1371/journal.pone.0000694)
Supplement: Figure S2 — Experimental design for gene silencing studies on vegetative medusae of the hydrozoan Eleutheria dichotoma. At day 0, each of the 10 vegetative i.e. budding medusae were placed in a Boveri dish and treated either with dsRNA (RNAi) or antisense morpholinos, or treated in different ways as controls (Control). After approximately one week, the first medusa buds are released from the parent medusa (circled and highlighted), which then start reproducing vegetatively. In this way, by the end of the experiment the total number of medusae increases to values between 90 and 160 medusae. The original 10 parent medusae at the beginning of the experiment thus represent some 6 to 11% of the final population. If one assumes that only medusa buds are transfected, the percentage of PAMs could be as high as 90% by the end of the experiment. (0.15 MB PDF) [file pone.0000694.s002.pdf]

Fig. S4

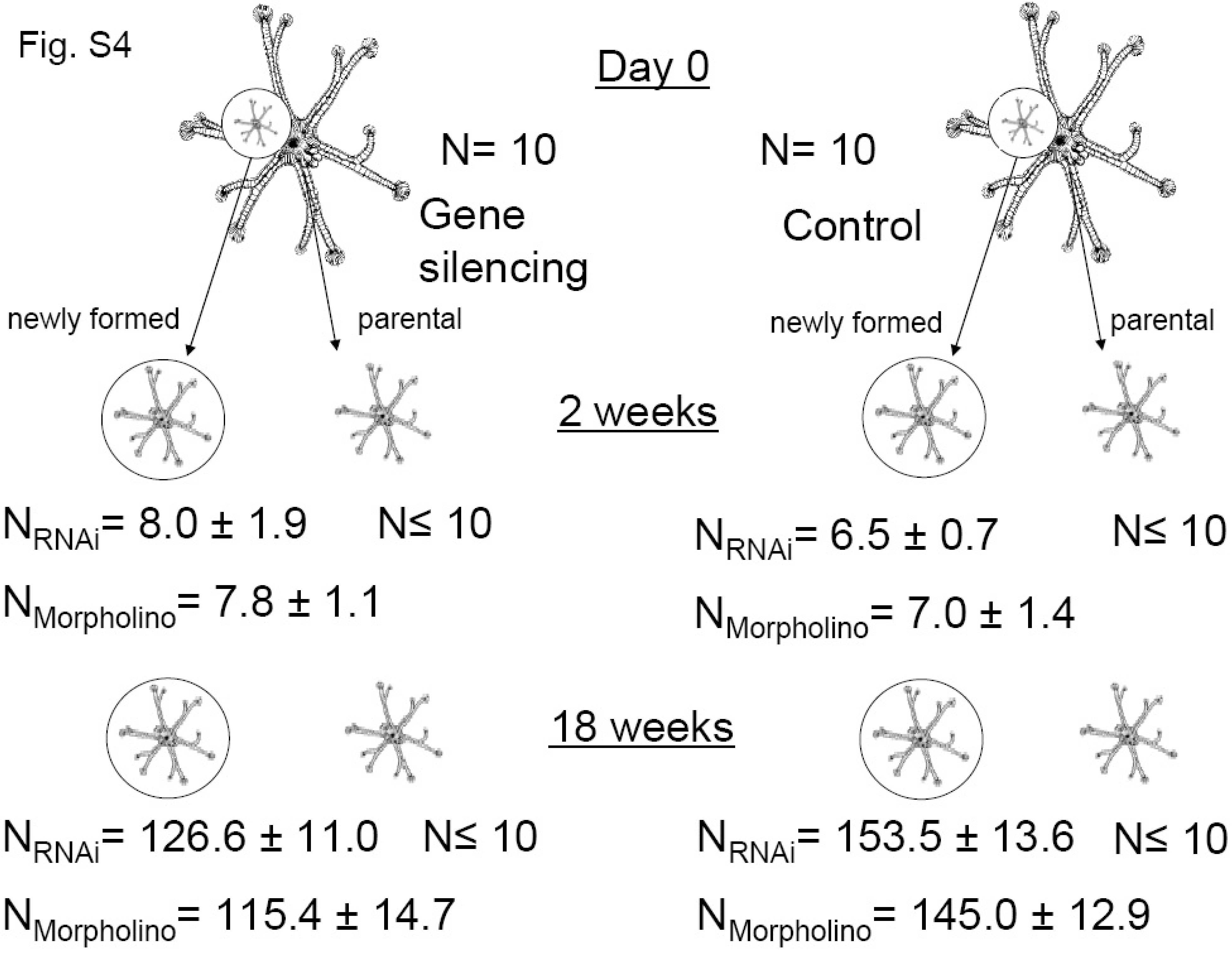

Day 0
N= 10
Gene silencing
N= 10
Control
newly formed
parental
newly formed
parental
2 weeks
N_{RNAi}= 8.0 ± 1.9
N≤ 10
$N_{Morpholino}$= 7.8 ± 1.1
N_{RNAi}= 6.5 ± 0.7
N≤ 10
$N_{Morpholino}$= 7.0 ± 1.4
18 weeks
N_{RNAi}= 126.6 ± 11.0
N≤ 10
$N_{Morpholino}$= 115.4 ± 14.7
N_{RNAi}= 153.5 ± 13.6
N≤ 10
$N_{Morpholino}$= 145.0 ± 12.9
