# Supplementary figures and images for: Changing Hydrozoan Bauplans by Silencing Hox-Like Genes
Source: PLoS One. 2007 Aug 1;2(8):e694. doi: 10.1371/journal.pone.0000694 (PMC1931613; doi:10.1371/journal.pone.0000694)

600bp →

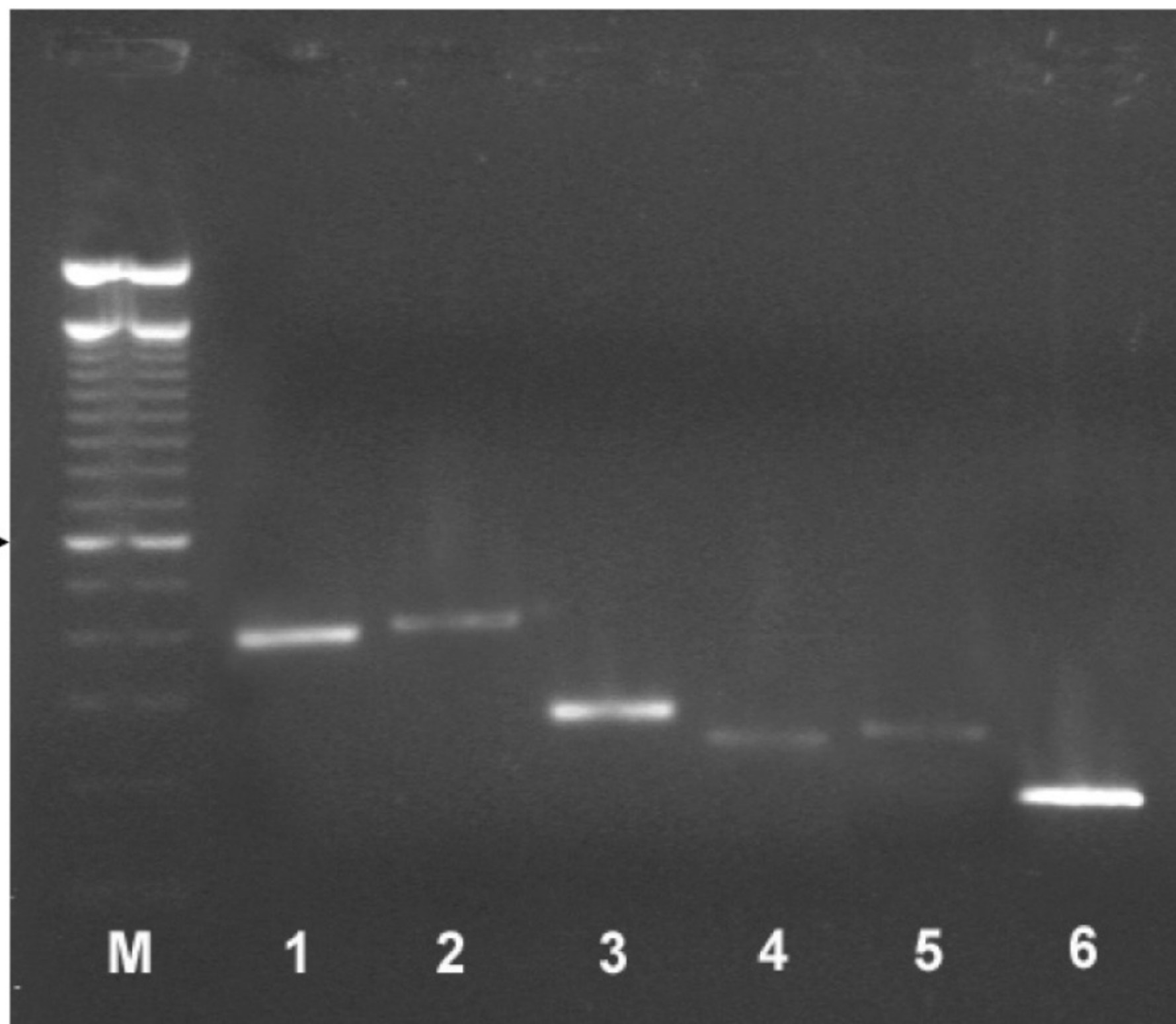

Supplement: Figure S3 — RT-PCR of all five Hox-like genes in Eleutheria dichotoma. Differential expression of Cnox-1 to Cnox-5 gene in Eleutheria dichotoma medusae. Shown are products of RT-PCR after 27 cycles of Cnox-1 (lane 1; 401bp), Cnox-2 (lane 2; 426bp), Cnox-3 (lane 3; 241bp), Cnox-4 (lane 4; 199bp), Cnox-5 (lane 5; 219bp) and Eleutheria-actin (lane 6; 152bp). Cnox-4 and Cnox-5 are only weakly expressed. (0.10 MB PDF) [file pone.0000694.s003.pdf]

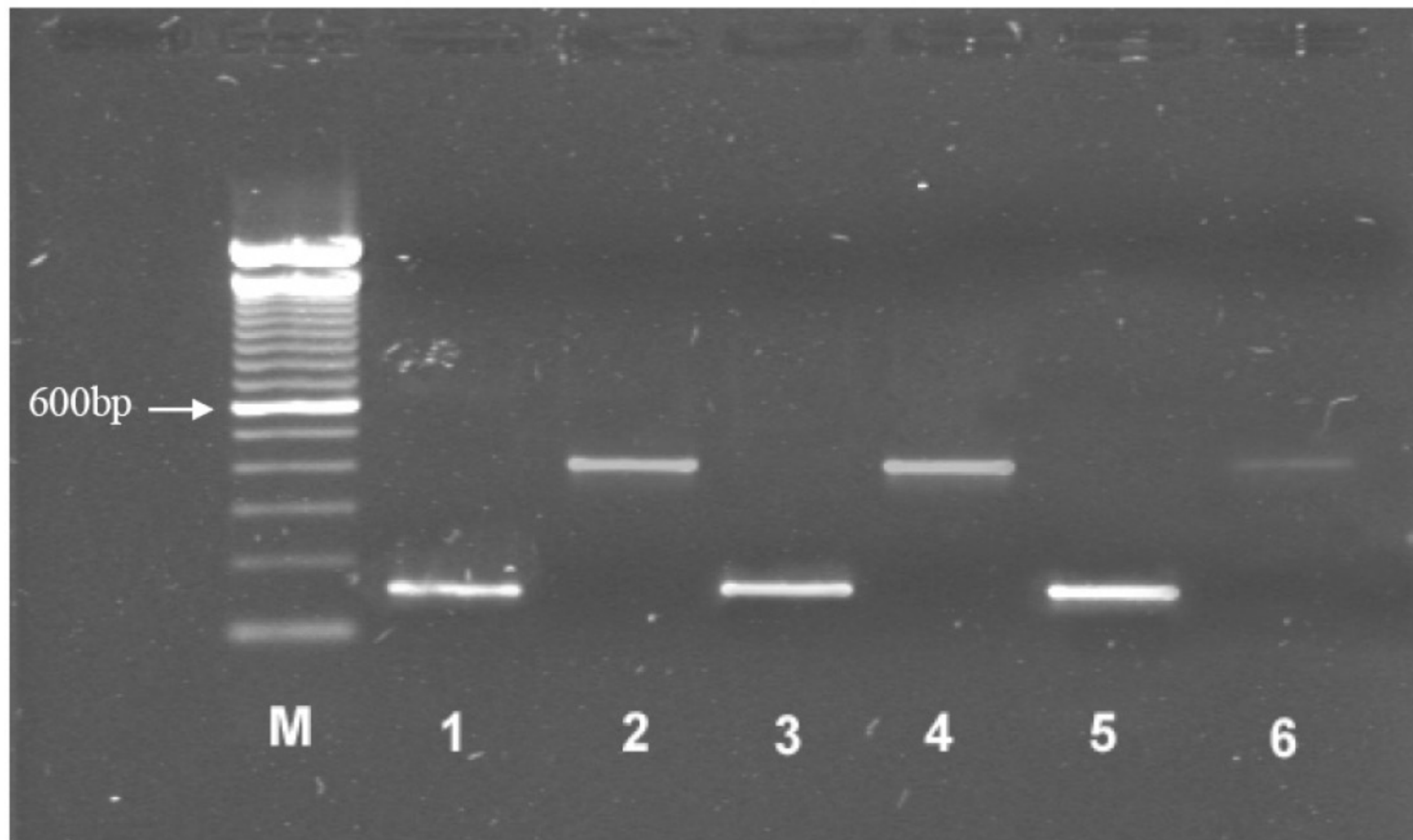

Supplement: Figure S4 — RT-PCR of Cnox-1-Ed after gene knock down. Transfection with dsRNA significantly reduces Cnox gene expression. RT-PCR products from untreated control animals are shown in lanes 1 and 2, from control dsRNA animals in lanes 3 and 4, and from Cnox-1 dsRNA-infected animals in lanes 5 and 6. Products in lanes 1, 3, and 5 are actin controls. Note the strong decline of Cnox product in lane 6 relative to lanes 2 and 4. Shown here is the example for Cnox-1 in Eleutheria dichotoma; RT-PCR controls look similar for all five Hox-like genes (data not shown). RT-PCR controls were taken as subsets from the transfected animals used in the gene silencing studies. (0.10 MB PDF) [file pone.0000694.s004.pdf]

A: RNAi

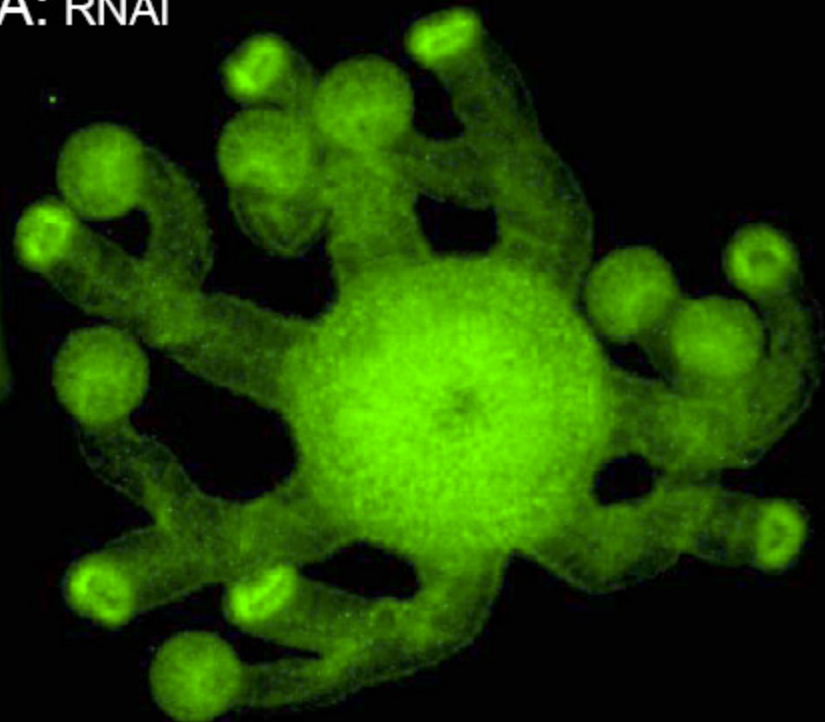

B: Morpholinos

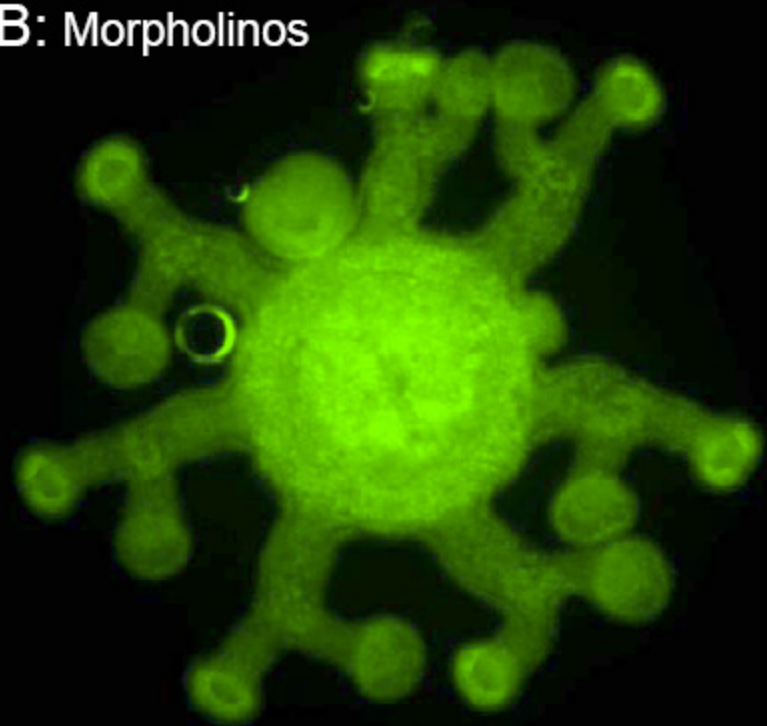

Supplement: Figure S5 — In vivo detection of fluorescein-labeled dsRNA (A) and morpholino oligomers (B) after transfection. (0.10 MB PDF) [file pone.0000694.s005.pdf]

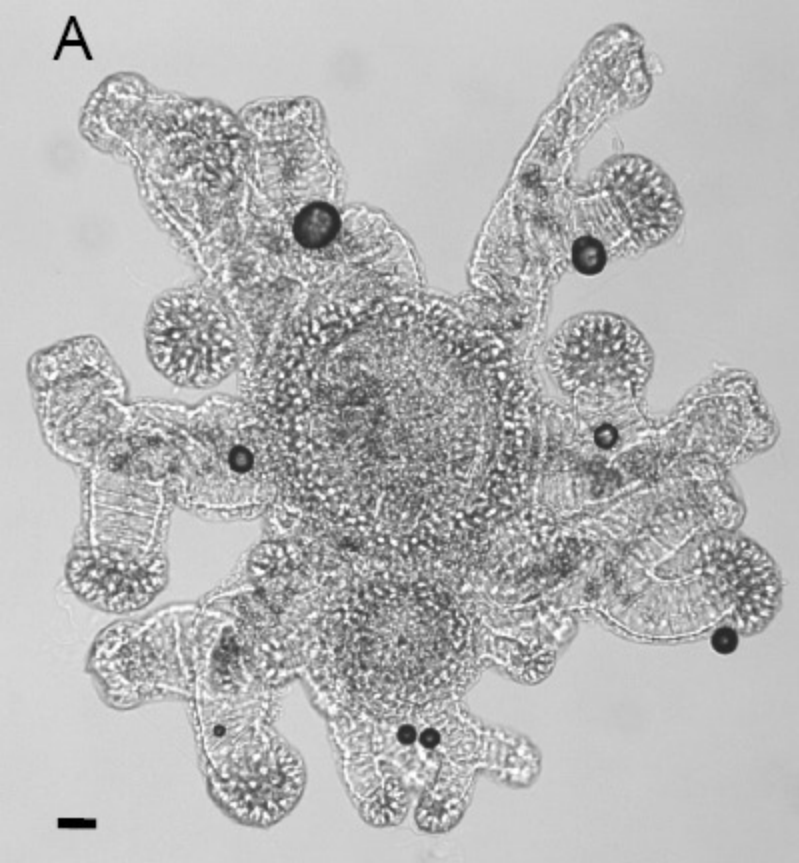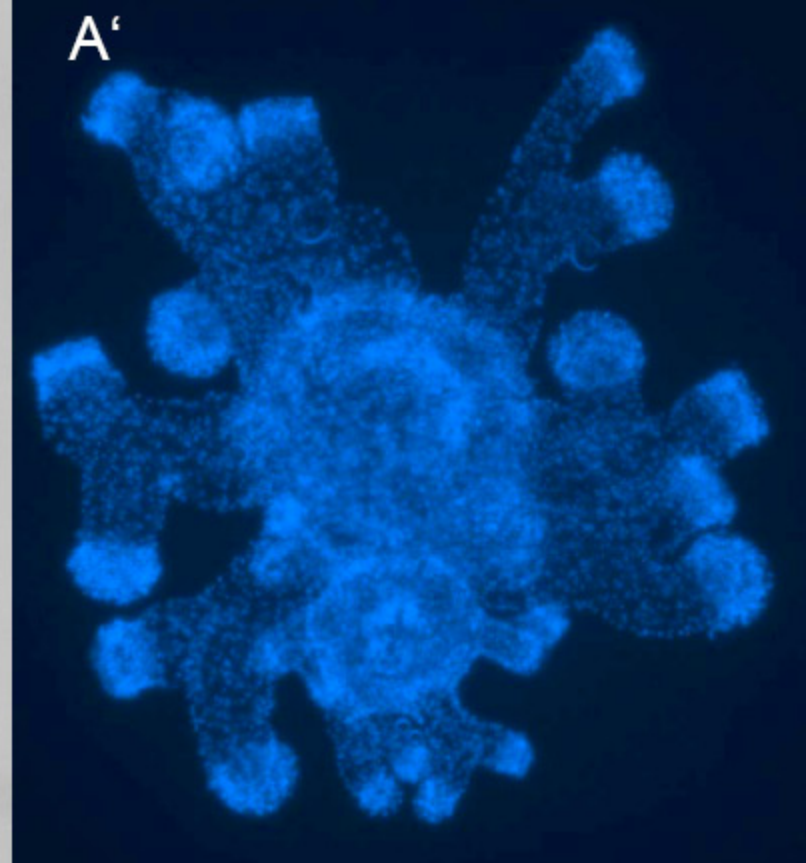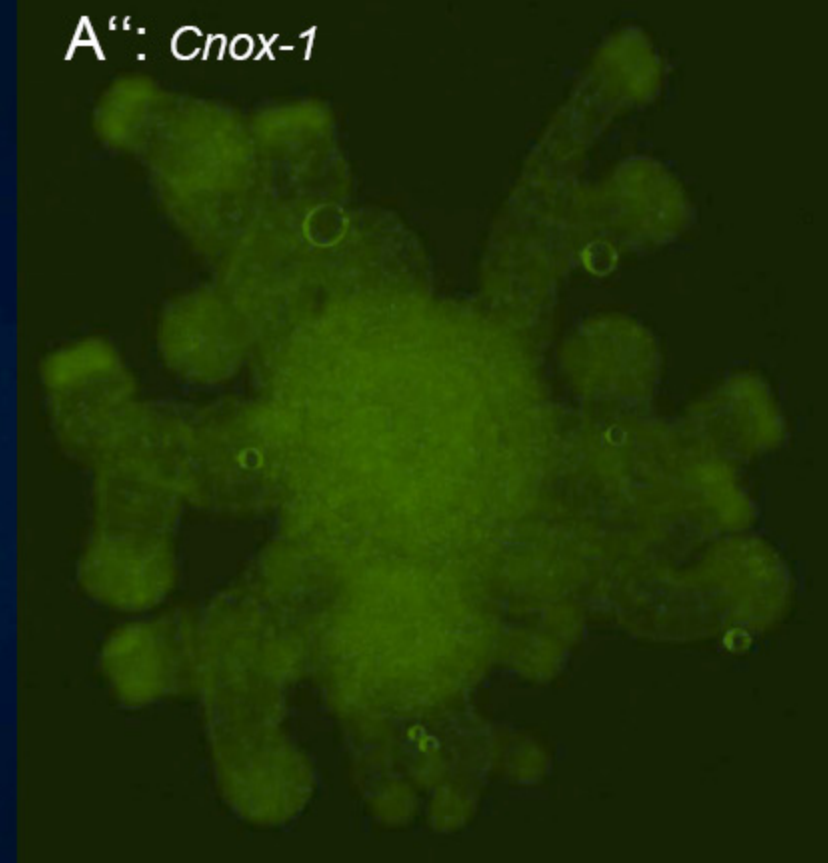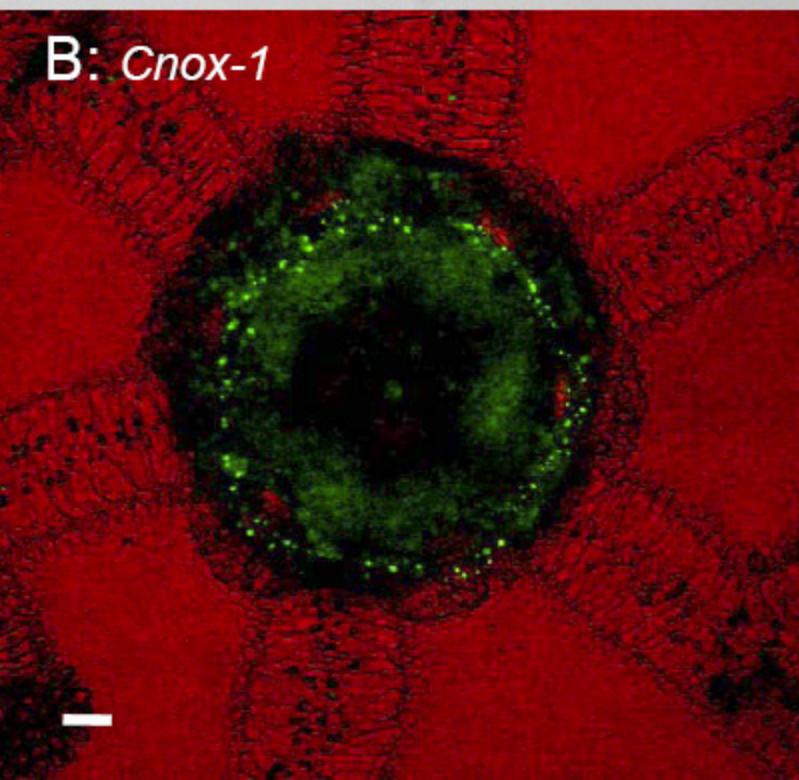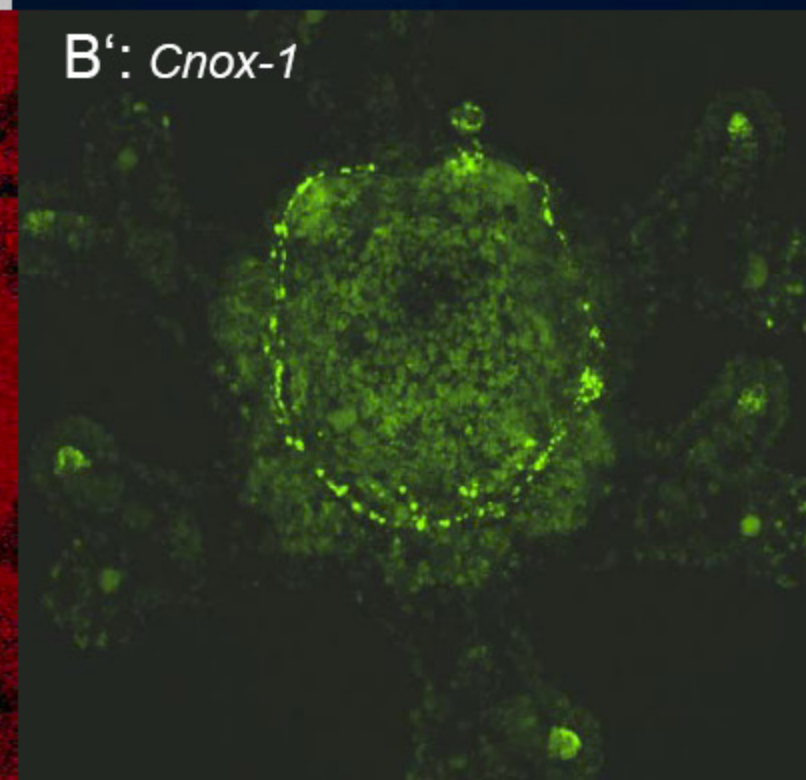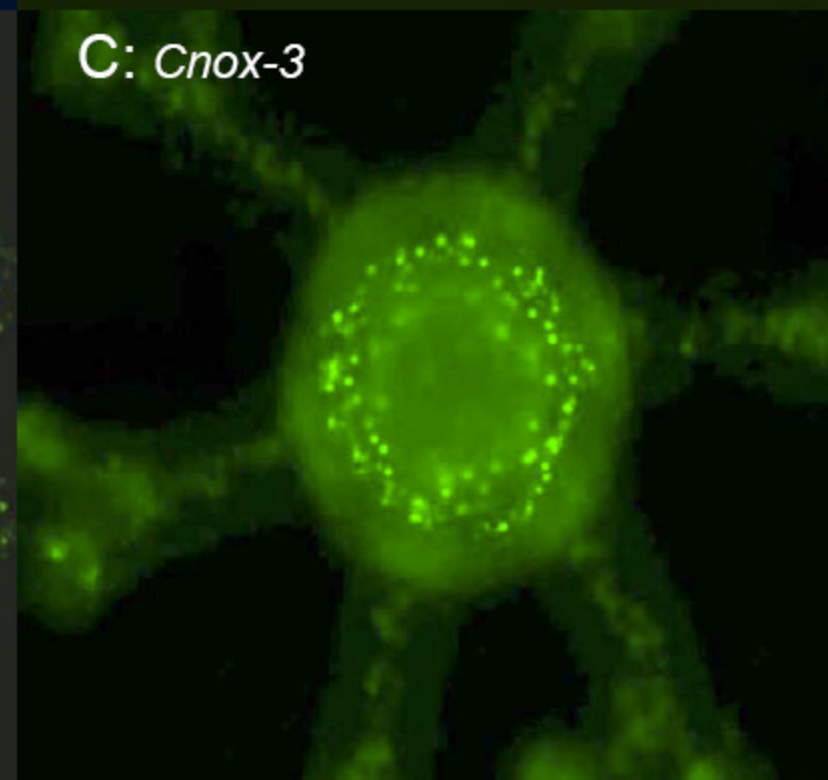

Supplement: Figure S6 — Target gene in situ hybridization of knock down animals. In situ hybridization of Cnox-1 gene in a Cnox-1 knock down animal (A'), in a RNAi-control animal (B), in a Morpholino-control animal (B') and Cnox-3 in situ hybridization in a Cnox-1 knock down animal. Medusa morphology is shown in light microscopy (A) and DAPI staining of the same medusa (A'). (0.29 MB PDF) [file pone.0000694.s006.pdf]
